# The step-like evolution of Arctic open water

**DOI:** 10.1038/s41598-018-35064-5

**Published:** 2018-11-15

**Authors:** Michael A. Goldstein, Amanda H. Lynch, Andras Zsom, Todd Arbetter, Andres Chang, Florence Fetterer

**Affiliations:** 1 Climate Change Research Center, University of New South Wales, Sydney, NSW 2052 Australia; 2 Finance Division, Babson College, Babson Park, MA 02457 USA; 3 Institute at Brown for Environment and Society, Brown University, Providence, RI 02912 USA; 4 Department of Earth, Environmental and Planetary Sciences, Brown University, Providence, RI 02912 USA; 5 Data Science Practice, Computing & Information Services, Brown University, Providence, RI 02912 USA; 6 National Snow and Ice Data Center, Cooperative Institute for Research in the Environmental Sciences, University of Colorado, Boulder, CO 80309 USA

## Abstract

September open water fraction in the Arctic is analyzed using the satellite era record of ice concentration (1979–2017). Evidence is presented that three breakpoints (shifts in the mean) occurred in the Pacific sector, with higher amounts of open water starting in 1989, 2002, and 2007. Breakpoints in the Atlantic sector record of open water are evident in 1971 in longer records, and around 2000 and 2011. Multiple breakpoints are also evident in the Canadian and Russian halves. Statistical models that use detected breakpoints of the Pacific and Atlantic sectors, as well as models with breakpoints in the Canadian and Russian halves and the Arctic as a whole, outperform linear trend models in fitting the data. From a physical standpoint, the results support the thesis that Arctic sea ice may have critical points beyond which a return to the previous state is less likely. From an analysis standpoint, the findings imply that de-meaning the data using the breakpoint means is less likely to cause spurious signals than employing a linear detrend.

## Introduction

In the most recent decade, summer minimum sea ice extent has retreated to levels not seen since the beginning of the satellite record^1^. The confluence of opportunity and risk at the retreating ice edge^2^ raises critical questions as to how well we observe and simulate Arctic ice area and extent. Conversely, how well do we describe the area covered by ocean within which ice, if present at all, is present in very low concentrations? In this context, the reconstruction of historical ice area records has been an important activity^3,4^. However, much of the focus has been upon hemispheric ice extent and concentration, whereas more critical to Arctic industrialization is the area and timing of seasonally navigable open water due to sea ice reductions. Industries such as freight, tourism, mining and commercial fishing may benefit from lower ice area, thickness, and concentration^5^. These activities have a critical reliance on shipping in the Arctic, which requires reduced or clear ice area over Arctic ocean sectors for transit navigability. However, because of the present low predictability of the navigable season, economic benefits have not accrued at the expected rate^6,7^. Low predictability has also led to ongoing safety and reliability concerns^8^. Indeed, even though summer sea ice is projected to have substantially retreated by as early as 2035, operators will face continued hazards from drift ice, icebergs and perhaps increased storminess^9,10^. While seasonally navigable open water is of course dependent on ship type, crew experience, and weather conditions, a reliable record of open water over recent decades provides a first-order understanding of how sea ice will affect Arctic economic development over coming decades.

Here, we examine the September sea ice area record across the Arctic Ocean and its peripheral seas for structural breaks. September is typically the average period that is used by climate researchers to document the advance of open water area over time^10–13^. There is a substantial literature that documents structural breaks in the global climate system, including attribution to mechanisms such as phase locking^14,15^. Further, steep transitions in the ice cover have been observed in smaller regions using a variety of data sets^16,17^. One hypothesis that has emerged is the idea of a low frequency propagating signal from the eastern to the western Arctic^18^. Despite this literature, to elucidate changes in ice variability, sea ice data continues to be de-trended linearly^11,19^. We find that de-trending using a change in means from identified breakpoints better describes the data by a number of measures.

While we examine the entire Arctic for breakpoints, a major interest in Arctic open water is the ability to transit from one side to another. Therefore, we look at two different set of halves separately, one set bounded by ocean, the other by land: the Atlantic and Pacific halves, which cover how sea ice might exit the Arctic Ocean and may be of more interest to ice scientists, and the Canadian and Russian halves, which consider possible transit routes from the lower Atlantic to the lower Pacific or vice versa, and so may be of more interest to shipping.

An essential first step is to interrogate the reliability of the data. During the satellite era, the data of record has been obtained from the Nimbus Scanning Multichannel Microwave Radiometer (SMMR: 1979–1987), the Defense Meteorological Satellite Program (DMSP) Special Sensor Microwave/Imager (SSM/I: 1987–2007) and the Special Sensor Microwave Imager/Sounder (SSMIS: 2008-present). Radiances are used to derive 25-km gridded sea ice concentration data products using either the NASA Team algorithm (data set ID: NSIDC-0051)^20,21^, which performs best with high-concentration multi-year wintertime ice, and the Bootstrap algorithm (data set ID: NSIDC-0079)^11,22^, which is considered more reliable in cases of surface melt or when concentrations are lower than about 40%^23,24^. Generally, no algorithm detects ice well when at very low concentrations (below 15%) and the low spatial resolution of the sensors limit the ability to distinguish an ice “edge”, however defined^25^. These records have been combined, accounting for the shortcomings in each, to produce an additional “merged” data product covering satellite era. Finally, the Climate Data Record (data set ID: G02202) uses the merged algorithm and ensures a temporally consistent analysis covering 1988–2017, although it ends in February 2017^26^.

None of these products can be considered to be independent, as they all derived from the same source measurement. As a result, we augment this record with the Hadley Centre Sea Ice and Sea Surface Temperature dataset (HadISST2.2)^24^. For the first half of the 20^th^ century this dataset relies largely on the Arctic and Southern Ocean Sea Ice Concentrations data set^27^ from the National Snow and Ice Data Center (NSIDC), which in turn makes use of sea ice charts from the Danish Meteorological Institute^28,29^. These charts are based on direct observations from the shoreline and ships^30^, and hence tend to overestimate ice extent in regions inaccessible by sea. Furthermore, these charts lack primary data for the time period 1940–1952, although this has been corrected as part of a substantive recent update^13^ that does not affect the results shown here. For the time period from 1960, sources expanded to include aerial surveys, observational re-analyses, operational ice charts, and from 1972, OSI-SAF (Ocean and Sea Ice Satellite Applications Facility) passive microwave retrievals, which is produced using SMMR, SSM/I, SSMIS, and (only in Version 1 of the data set) EMSR from 1972–1979. We show this data from 1953 for context, but our analysis focuses on the period from 1960 onwards, and particularly the satellite era. We also examined operational ice charts from the National Ice Center (Washington, DC) which are produced on a weekly to biweekly basis using all available forms of remotely sensed sea ice data, including the passive microwave data, visible and near-infrared products such as MODIS, and synthetic aperture radar such as RADARSAT^31^. Here, we use the ice extent from the 1972–2007 archived product^32^ for the last chart produced in September of each year. In processing the ice chart data for this study, any grid cell with ice concentration less than 15% was set to open water to be consistent with the satellite retrievals.

## Results

### Differences in detected ice area

**Figure 1:**
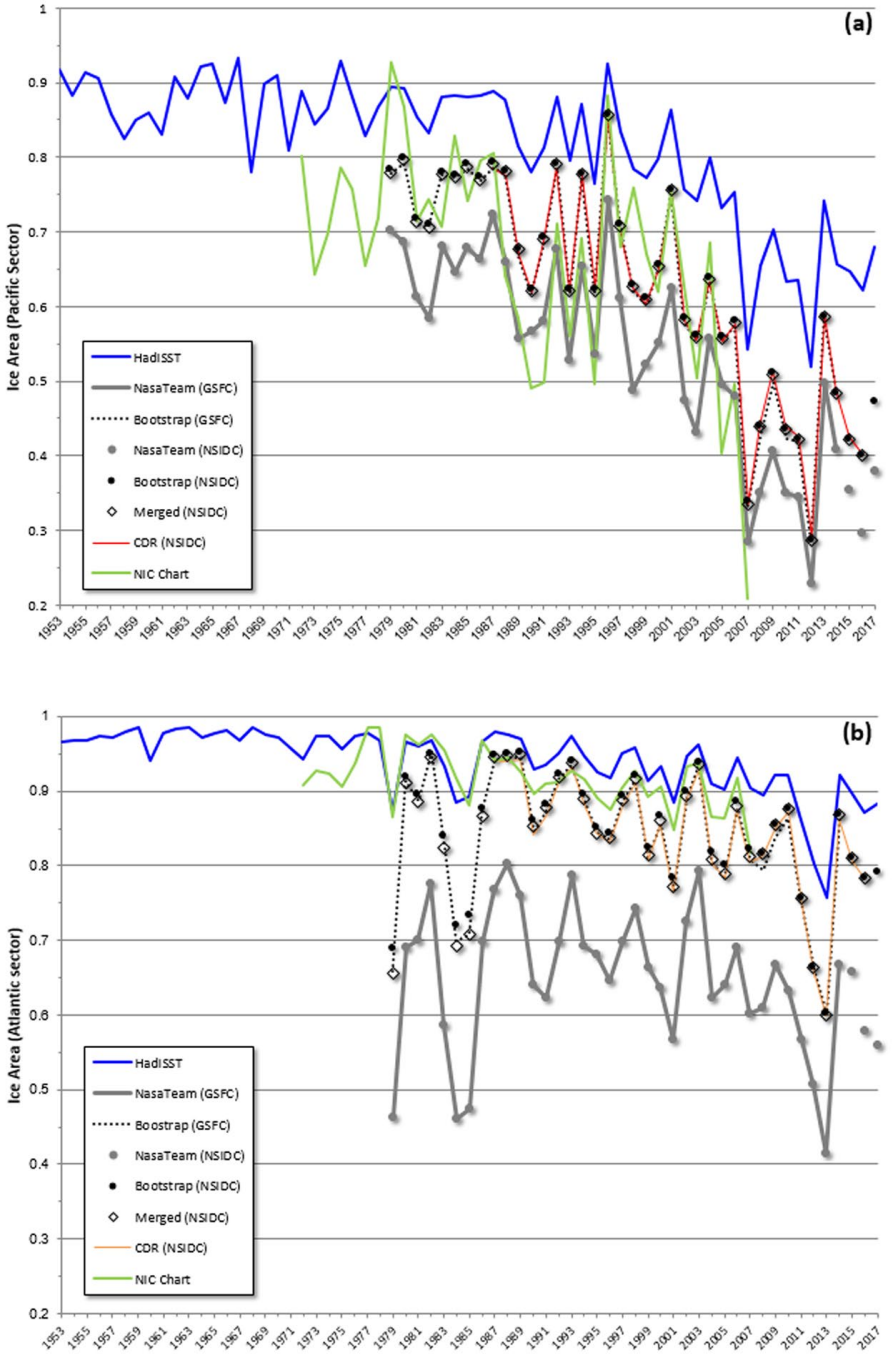
September ice area for (**a**) the Pacific sector (100^o^E-100^o^W) and (**b**) the Atlantic sector (100^o^W-100^o^E), 1953–2017. Blue line: HadISST (1953–2017). Grey line: NASA Team (GSFC). Black dotted line: NASA Bootstrap (GSFC). Grey circle: NASA Team (NSIDC). Black circle: NASA Bootstrap (NSIDC). Black diamond: Merged product (NSIDC). Orange line: CDR (1988–2017). Green line: NIC Charts (1972–2007).

The fraction of a grid cell covered in ice, or ice area, is calculated for the Atlantic and Pacific sectors for September for each of the satellite derived products, as well as the HadISST product and the operational ice charts (see Methods). September is at the end of the melt season and so tends to have the lowest ice area^9^ as well as the largest downward interannual trend^19^. The records show some substantial differences across the records (Fig. 1). The Bootstrap algorithm results in systematically lower open water area than the NASA Team algorithm. Within the microwave radiance derived data sets, discrepancies arise from small differences between the algorithms used to process brightness temperatures to ice areas, small corrections in the brightness temperatures themselves, and to a lesser extent the characteristics of computational resources used to generate the datasets^26^. The differences are small but not insignificant^21,33,34^. The CDR and merged products are obtained by comparing the ice areas derived by the NASA Team and Bootstrap algorithms and choosing the higher concentration of the two, along with other improvements^26^. For our study, the CDR generally follows the Bootstrap curve, as does the merged product. In the case of HADISST and NIC ice charts, the discrepancies are larger, and arise from the use of additional source data such as aircraft observations and synthetic aperture radar. The HadISST record shows systematically higher ice area than any of the satellite-derived records, primarily because the HadISST product uses 1 degree grid spacing rather than the 25 km used in the satellite-derived records; this lower resolution results in few grid cells reporting below 15% ice area over the entire cell, resulting in a higher area average. In addition, the HadISST product uses the NIC charts from 1995 “as the representation of the ‘true state’”^24^. The NIC charts in the Atlantic sector during high ice years generally report more ice cover than the satellite-derived records. Differences of the magnitude shown here are large enough to affect seasonal forecast skill^35^, causing disparities of the order of 2 °C in a five month forecast when used for initialization.

### Abrupt shifts in sea ice coverage

**Figure 2:**
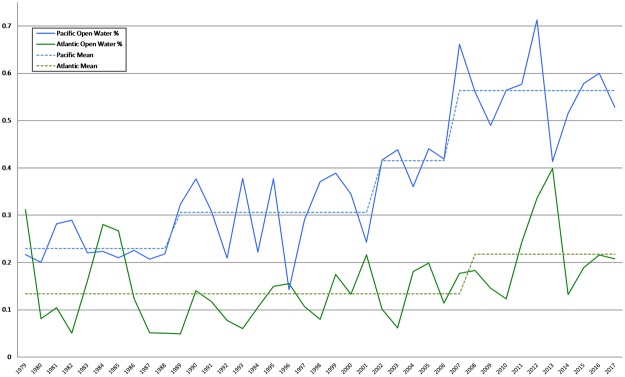
September open water area percentage from the NSIDC NASA Team data record for the Pacific (blue) and Atlantic (green) sectors. Dashed lines show the breakpoint model using the breakpoint years detected in Table 1, Panel A.

The data in Fig. 1 for the Pacific sector suggests that the area of ice cover has decreased for some time, while the data for the Atlantic sector is less indicative. While some have suggested the data is characterized by a linear trend^36^ and others have detrended the ice record linearly in order to understand ice retreat and interannual variability^9,19^, the data for the Pacific sector suggests there may be a shift in the mean (that is, a structural change or breakpoint) in the data; if so, a linear trend is not the correct functional form. To investigate this, we apply a breakpoint analysis designed to allow early determination of breakpoints (see Methods) to the sea ice record. We also examine open water, which is determined as the area average of one minus the fraction of ice cover in each grid cell within the ice pack (Fig. 2). Open water is related to navigability, and hence can provide a policy relevant perspective. While magnitude varies across the microwave brightness temperature derived ice area records, the NASA Team (NSIDC) record is representative in its trends and variability. Overall, the record reveals key structural shifts in the mean. These shifts are robust to the different satellite analysis algorithms described above (not shown) but are limited geographically (Table 1, Panel A). The results suggest that breakpoints in ice regimes have already occurred in the record, although artifacts arising from the brevity of temporally consistent records cannot conclusively be ruled out.

**Table 1:** Mean Square Error (MSE), Adjusted R^2^ (Adj R^2^) and Bayesian Information Criterion (BIC) for linear and breakpoint models, along with the breakpoint years for the Atlantic, Pacific, Canadian, and Russian sectors, and for the whole Arctic.

Ocean	Linear			Breakpoint			Breakpoint Years					
	MSE	Adj R^2^	BIC	MSE	Adj R^2^	BIC						
**Panel A: NSIDC-NASA Team**												
Pacific	1.44E + 11	0.73	1006	1.01E + 11	0.81	995	1989		2002		2007	
Atlantic	2.64E + 10	0.46	940	2.88E + 10	0.41	943					2008	
Canadian	5.29E + 10	0.62	967	3.72E + 10	0.74	955		1998			2007	
Russian	8.46E + 10	0.73	985	5.70E + 10	0.82	971	1990			2005		
ALL Arctic	1.65E + 11	0.82	1011	9.83E + 10	0.89	994	1989	1998			2007	
**Panel B: HAD-Truncated**												
Pacific	2.47E+ 11	0.72	1027	1.63E + 11	0.81	1014	1989		2002		2007	
Atlantic	5.27E + 10	0.60	967	4.81E + 10	0.64	965		2000				2011
Canadian	7.97E + 10	0.71	983	5.12E + 10	0.81	967		1998			2007	
Russian	1.73E + 11	0.70	1013	1.57E + 11	0.73	1010				2005		
ALL Arctic	3.40E + 11	0.78	1040	3.00E + 11	0.80	1036			2002		2007	
**Panel C: HAD-Full**												
Pacific	3.55E + 11	0.56	1547	1.91E + 11	0.77	1513	1989		2002		2007	
Atlantic	6.02E + 10	0.61	1444	4.97E + 10	0.67	1435	1971	2000				2011
Canadian	1.34E + 11	0.46	1490	6.49E + 10	0.74	1449		1998			2007	
Russian	1.97E + 11	0.68	1513	1.14E + 11	0.81	1483	1971	1990		2005		
ALL Arctic	5.21E + 11	0.66	1569	2.96E + 11	0.80	1537		1998			2007	

The data for Panel A is from the NASA Team (NSIDC) open water record for 1979–2017; Panel B, the HadISST record for 1979–2017; and Panel C, the HadISST record for 1953–2017.

### Abrupt shifts or linear trend

In order to address whether these breakpoints provide an improved model for sea ice area time series, we commence with regressions testing for a linear trend (see Methods). We apply this analysis to the NASA Team (NSIDC) record in Fig. 2, as well as the HadISST record for a truncated time series equivalent to the satellite record, and for the HadISST time series commencing in 1960.

We start by examining trends in open water percentage using data from 1979 to 2017. It is apparent that there is a weak linear trend (2% per decade, adjusted R^2^ = 0.09) in the Atlantic sector but a large and significant linear trend (11% per decade, adjusted R^2^ = 0.69) in the Pacific sector (numbers quoted for the NASA Team (NSIDC) record).

To investigate whether breakpoints might better describe the data than a linear trend, we compare the results for linear trend models with models where breakpoints are estimated (see Methods). The Pacific sector had three major breakpoints: 1988/89, 2001/02, and 2006/07 (Table 1), regardless of which dataset is used. One or more of these breakpoints was found in each of the six permutations, so these breakpoints are generally robust to dataset or model inputs. Notably, these three breakpoints provide a lower mean squared error (MSE) and Bayesian information criterion (BIC) than the linear trend model. For the full HadISST dataset, a regression model with these three breakpoints had a MSE of 1.91E + 11, a BIC of 1513, and an adjusted R^2^ of 0.77, while the linear trend model had a notably higher MSE of 3.55E + 11 and BIC of 1547 and a lower adjusted R^2^ of 0.56 (Table 1, Panel C). Similar results (Table 1) were found for the NASA Team (NSIDC) data (1979–2017, hereinafter called NSIDC-NASA Team), a truncated version of the HadISST data (using only data from 1979–2017, hereinafter called HAD-Truncated) as well as the full HadISST record from 1960 to 2017 (hereinafter called HAD-Full). The Pacific is better characterized by four mean levels with a series of three abrupt transitions rather than a linear trend (Fig. 2). For the HAD-Full dataset, the Atlantic results suggest good support for breakpoints in 1970/71, 1999/2000, and 2010/11. A model with these breakpoints has an MSE (4.97E + 10) that is 17% lower than the MSE for the linear trend model (6.02E + 10) as well as a lower BIC and a higher adjusted R^2^. Similar breakpoints at 1999/2000 and 2010/11were found using the HAD-Truncated data. Using the NSIDC-NASA Team data, a single breakpoints was found at 2007/08; the Atlantic NSIDC-NASA Team was the only one in Table 1 where the breakpoint did marginally worse than the linear trend.

All six permutations of the breakpoint algorithm on the Canadian half found breakpoints in the HAD-Full record at 1997/98 and 2006/07 (Table 1). A model with breakpoints fit the data notably better than the linear model. For example, a model on the HAD data with only two breakpoints at 1997/98 and at 2006/07 have an MSE (6.49E + 10) that is 52% smaller than the MSE of the linear model (1.34E + 11) and a smaller BIC, and the adjusted R^2^ for the breakpoint model (0.74) is notably larger than that for the linear model (0.46). All permutations for Canadian half data do better than a linear model for the HAD-Full, HAD-Truncated, and NSIDC-NASA Team datasets. For the Russian half, the HAD-Full results suggest breakpoints at 1970/71, 1989/90, and 2004/05. The all six permutations for the shorter results for NSIDC-NASA Team suggest breakpoints at 1989/1990 and 2004/2005, and all six permutations of the HAD-Truncated strongly suggest a breakpoint at 2004/05. In all cases for all six permutations, the HAD-Full, HAD-Truncated, and NSIDC-NASA Team results for the Russian half suggest a lower MSE, a lower BIC, and a higher adjusted R^2^ than the linear trend.

Given the differences in the results for the sectors above, it is unclear if the entire Arctic should be considered as a whole. Even so, for the entire Arctic, for the HAD-Full data, all six permutations have lower MSEs, lower BICs, and higher adjusted R^2^ than the linear trend model. Generally, there is support for a breakpoint at 1998/1999 and 2006/07, as well as some support for breakpoints at 1989/90 and 2001/02. All of the six permutations for the NSIDC-NASA Team and 4 of 6 for HAD-Truncated have lower MSEs and BICs and higher adjusted R^2^ than the linear model.

The short record of satellite-derived time series prior to 1988 is a limitation of this model. The NIC chart-derived time series from 1972–2007 was tested for the Pacific and the Atlantic sectors. Because this record stops in 2007, only one breakpoint, in 1988, could be assessed. It was found that both models – a single trend variable or one mean break (1972 to 1988, 1989 to 2007) – were insignificant in combination, and equally valid when tested separately. The adjusted R^2^ for the Pacific was around 0.29 and for the Atlantic was around 0.25. That is, either a linear trend or a structural break in 1988 was a similarly legitimate model of the NIC chart time series. No other breakpoints were identified in this record in either sector.

The HadISST record is not temporally uniform, and has a lower spatial resolution than the satellite derived records. In the Pacific sector, the HadISST time series reflects the NSIDC-NASA Team results.

## Discussion

These findings suggest caution must be employed when seeking to understand trends in Arctic ice cover over the satellite era and to reproduce these trends in operational and climate models. Further, it calls into question the practice of characterizing the record using linear trends. A model of abrupt shifts in September open water fraction is likely to have a number of drivers that interact in complex ways. First, it is possible that non-physical artifacts may exist in the satellite record. Several authors have noted that small artifacts remain in the satellite-derived records, associated with sensor and orbit changeovers, but all suggest that these differences are below the sensitivity of the instrument^37–39^. That said, it is known that there were some errors in the data stream in the first months of the SSM/I instrument^40^. While this may have some impact on the structural shift detectable in 1988, given that other breakpoints were detected there are likely to be other, physical, factors that are important.

Structural changes in the ice cover record have been suggested previously. In the eastern Bering Sea, a late 70′s regime shift in the long term sea ice cover record was associated with a 50 to 70 year oscillation in the North Pacific^41,42^. Shifts in the magnitude of the decreasing trend in Arctic sea ice extent around 2005–2007 were also detected^33^. Unlike the method employed here, which is designed for lower data frequency as in the satellite record, others have employed algorithms on individual gridpoints assuming a linear trend on LOESS smoothed data, which requires large, densely sampled data and reduces the effect of outliers and thus may understate or miss transition points, and only consider one breakpoint per location^43^.

**Figure 3:**
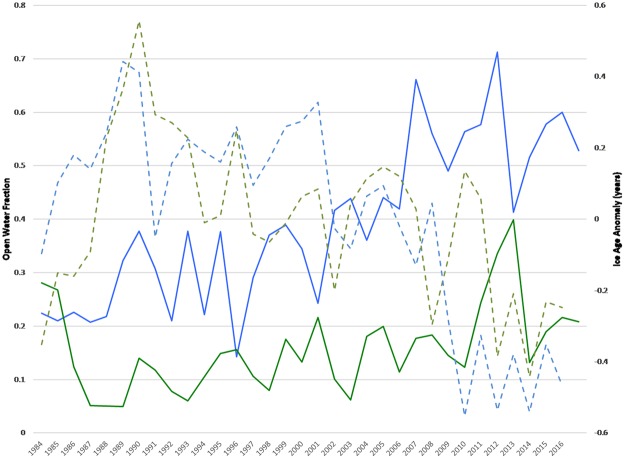
Ice age anomaly (dashed) and open water (solid) derived from EASE-Grid Sea Ice Age, Version 3 and NSIDC-NASA Team from 1984–2017 for the Pacific (blue) and Atlantic (green).

The hypothesis that the ice volume reaches a critical threshold allowing subsequent rapid melting is one that has been identified in model studies^44^. In future climate scenarios, abrupt changes in September ice extent and thickness that are characterized by thermodynamically driven doubling of ice loss for periods of around five years, in seven of sixteen models participating in the A1B scenario of the IPCC-AR4, have been detected. Ice age is a useful, though not perfect, proxy for ice thickness. Figure 3 shows anomalies of perennial ice age derived from EASE-Grid Sea Ice Age, Version 3^45^ for week 37 (that is, late September), where anomaly is defined relative to a base period of 1984 through 2016. Note that ice age anomalies increase rapidly in the first 6 years of the record–this is likely an artifact of the data product’s initialization procedure. After 1990, multiyear ice with concomitant positive anomalies in both the Pacific and the Atlantic dwindles, particularly after the successive record low summers of 2007 and 2012, with low anomalies in 2008 (Atlantic) and 2010 (Pacific) and falling dramatically lower in 2012 and 2014 in both the Pacific and the Atlantic. Very little multiyear ice remains; in fact, since the algorithm records only the oldest ice in the grid cell, the amount of old ice in recent data may be overestimated. Nevertheless, there is a strong negative relationship between the ice age record and the open water area (also shown in Fig. 3). These records are correlated at −0.71 in the Pacific sector and −0.48 in the Atlantic sector. Using ice age data from 1984–2016, a model for ice age in the Pacific sector with a constant and dummy variables for the 1988/89, 2001/02, and 2006/2007 breakpoints noted in Table 1, has a lower MSE (0.71), a lower BIC (−4.75) and a higher adjusted R^2^ (0.73) than the linear trend model (MSE = 1.20; BIC = 10.29, adjusted R^2^ = 0.54). In the Atlantic sector, the linear trend model (MSE = 0.764, BIC = −4.68, adjusted R^2^ = 0.13) does worse than a model with a breakpoint at 2007/08 (MSE = 0.665, BIC = −9.28, adjusted R^2^ = 0.25), perhaps because the Atlantic provides an escape route for Arctic ice.

Because of the initialization of the ice age record, it is not possible to ascribe a structural shift unambiguously in 1990. However, it appears that an abrupt increase in open water area can be associated with a concomitant abrupt decrease in ice age across the Arctic. Further, it is possible that 2007 and 2012 represents something of a critical point for the sea ice, particularly in the Pacific.

A long-term, high quality and temporally consistent record of Arctic open water remains an elusive goal^46^. That said, there is much insight that can be derived from a careful analysis of all of the available data. This assessment of satellite-derived, operational, and climatological time series suggests that Arctic sea ice behavior is highly non-linear, and critical points in ice cover that have been postulated as arising from strong ice-albedo feedback processes may have already occurred. We have demonstrated that, certainly in the Pacific sector and perhaps for the Atlantic sector, as well as for the Canadian and Russian halves, sea ice area is better characterized by a series of structural shifts, in 2007 and in near the turn of the century in 1998 and/or 2002, rather than a linear trend. This model is robust to the algorithm used to derive ice concentration from satellite passive microwave brightness temperatures, although these algorithms demonstrate significant differences in magnitude. The physical nature of these structural shifts is supported by analysis of quasi-independent records, such as the operational ice charts. The concomitant shifts in the ice age product suggests a potential thermodynamic feedback mechanism. That said, the processes associated with these shifts can only be tested independently using a modeling approach, which will be the subject of future work.

## Methods

### Ice index

For each satellite data record and each year in the record, a mean September sea ice area within a Pacific and an Atlantic sector is determined. The Pacific sector is bounded by 100^o^W, 100^o^E, 66.6^o^N, and 84.5^o^N. For the Atlantic sector boundaries are 100^o^E, 100^o^W, 66.56^o^N and 84.5^o^N. The Russian sector is bounded by 170^o^W, 0^o^E, 66.56^o^N, and 84.5^o^N. For the Canadian sector boundaries are 0^o^E, 170^o^W, 66.56^o^N and 84.5^o^N. Within each sector and for each day, the number of grid cells for which the ice concentration is less than 15% is tallied and the area covered by those cells is computed. A monthly average is then computed from these daily areas. HadISST data and National Ice Center sea ice chart data were processed in the same way, although the National Ice Center charts were not available as daily data.

### Breakpoint Analysis

We use a sequential algorithm^47^ that can find multiple breakpoints and which allows for the choice of different lengths (L) of the number of years and significance levels (p) in determining breakpoints. We vary our number of year (length) inputs from 5 to 10 as inputs into the breakpoint methodology, resulting in six permutations of breakpoints. For the results reported in the paper, we used p = 0.05; for robustness, we also ran the tests using breakpoints based on p = 0.10 and got substantively the same results, and so did not report them here. We then apply this methodology and these six permutations to the Atlantic and Pacific sectors, the Canadian and Russian sectors, and the entire Arctic.

For the statistical tests to compare the accuracy of the breakpoints versus a linear trend, the following models were compared. The linear trend model takes the form:

1 $${Y}_{t}=\alpha +\beta \cdot t+{{\epsilon }}_{t}$$

where *Y*_*t*_ is the value of the sea ice concentration or ice age for year *t*, and *t* is the time in years (creating a linear trend). For the breakpoint analysis, a model was created using the breakpoints. The generalized form of the model is

2 $${Y}_{t}=\sum _{t=0}^{t=N}\{mean({Y}_{{t}_{i}:{t}_{i+1}})\ast {d}_{{t}_{i}:{t}_{i+1}}\}+{{\epsilon }}_{t}$$

where *t* = 0 and *t* = N are the first and last years in the dataset, *t*_*i*_ and *t*_*i*+*1*_ are the break points returned by the sequential algorithm, $mean({Y}_{{t}_{i}:{t}_{i+1}})$ is the mean sea ice concentration between *t*_*i*_ and, and *t*_*i*+*1*_ (with the first mean starting at t = 0 and continuing up to but not including the first breakpoint, and the last mean starting with the last breakpoint year and continuing to t = N), and ${d}_{{t}_{i}:{t}_{i+1}}$ is a dummy variable that equals 1 between *t*_*i*_ and *t*_*i*+*1*_ and 0 otherwise.

This form can be translated into a regression, either with or without an intercept. For example, for a model of the Pacific with three breakpoints, such as p = 0.5 and L = 7, where the breakpoints were 1989, 2002, and 2007, a three breakpoint model without an intercept took the form:

3 $$\begin{array}{rcl}{y}_{t} & = & {\beta }_{1}\ast {d}_{1}+\,{\beta }_{2}\ast {d}_{2}+{\beta }_{3}\ast {d}_{3}+{\beta }_{4}\ast {d}_{4}\\  &  & +\,{\varepsilon }_{t}\,where\,\{\begin{array}{l}{d}_{1}=1\,if\,\,\,\,\,\,\,t\le 1988\,and\,0\,otherwise\\ {d}_{2}=1\,if\,1989\le t\le 2001\,and\,0\,otherwise\,\\ {d}_{3}=1\,if\,2002\le t\le 2006\,and\,0\,otherwise\\ {d}_{4}=1\,if\,2007\le t\le 2014\,and\,0\,otherwise\end{array}\}\end{array}$$

This regression in (3) above would return the means of the four periods as the coefficients on the dummy [0, 1] variables. In addition, the regression without an intercept (3) is functionally equivalent to a regression with an intercept and three dummy variables:

4 $${y}_{t}=\alpha +{\beta }_{2}\ast {d}_{2}+{\beta }_{3}\ast {d}_{3}+{\beta }_{4}\ast {d}_{4}+{{\epsilon }}_{t}\,$$

where *d*_2_, *d*_3_, and *d*_4_ are defined as in regression (3) above.

The mean squared error (MSE) and Bayesian information criterion (BIC) were calculated for the linear trend model and each of the six models created by the six permutations of the Rodionov (2004) breakpoint algorithm. Note that both the regression without an intercept (3) and with an intercept (4) will have the same MSE and BIC, but the adjusted R^2^ is only well-defined for regression (4). The goodness-of-fit of each of the six breakpoint models was compared with the linear model using MSE, BIC, and adjusted R^2^. Breakpoints that were found in multiple permutations were considered more robust to parameter estimates. Table 1 provides the best fit (lowest MSE and BIC; highest adjusted R^2^ breakpoints) of the six examined for each area and dataset; the full set is found in Supplement Table 1.

## Electronic supplementary material

Supplementary Information

## References

[1] Fetterer, F., Knowles, K., Meier, W. & Savoie, M. *Sea Ice Index*. Boulder, Colorado USA: National Snow and Ice Data Center, updated daily, 10.7265/N5QJ7F7W (2002).

[2] Lei RB, Xie HJ, Wang J, Lepparanta M, Jonsdottir I, Zhang ZH (2015). Changes in sea ice conditions along the Arctic Northeast Passage from 1979 to 2012. Cold Regions Science and Technology.

[3] Polyak L (2010). History of sea ice in the Arctic. Quatern. Sci. Rev..

[4] Rogers TS, Walsh JE, Rupp TS, Brigham LW, Sfraga. M (2013). Future Arctic marine access: analysis and evaluation of observations, models, and projections of sea ice. The Cryosphere.

[5] Stephenson SR, Smith LC, Brigham LW, Agnew JA (2013). Projected 21st-century changes to Arctic marine access. Climatic Change.

[6] Krupnik, I. & Jolly, D. *The Earth Is Faster Now: Indigenous Observations of Arctic Environmental Change*. (Arctic Research Consortium of the U.S., Fairbanks, AK 2002), 356pp.

[7] AMAP. Snow, water, ice, and permafrost in the Arctic (SWIPA). Arctic Monitoring and Assessment Programme (AMAP). Oslo (2011).

[8] Liu M, Kronbak. J (2010). The potential economic viability of using the Northern Sea Route (NSR) as an alternative route between Asia and Europe. Journal of Transport Geography.

[9] Parkinson CL, Comiso JC (2013). On the 2012 record low Arctic sea ice cover: Combined impact of preconditioning and an August storm. Geophys. Res. Lett..

[10] Koyama T, Stroeve J, Cassano J, Crawford A (2017). Sea Ice Loss and Arctic Cyclone Activity from 1979 to 2014. J. Climate.

[11] Serreze MC, Stroeve J (2015). Arctic sea ice trends, variability and implications for seasonal ice forecasting. Phil. Trans.R. Soc. A.

[12] Polyakov IV (2017). Greater role for Atlantic inflows on sea-ice loss in the Eurasian Basin of the Arctic. Ocean. Science.

[13] Walsh JE, Fetterer F, Scott Stewart J, Chapman WL (2017). A database for depicting Arctic sea ice variations back to 1850. Geogr Rev.

[14] Swanson KL, Tsonis AA (2009). Has the climate recently shifted?. Geophys. Res. Lett..

[15] Douglass DH (2010). Topology of Earth’s climate indices and phase-locked states. Physics Letters A.

[16] Liu J, Curry JA, Hu Y (2004). Recent Arctic Sea Ice Variability: Connections to the Arctic Oscillation and the ENSO. Geophys. Res. Lett..

[17] Mahoney AR, Barry RG, Smolyanitsky V, Fetterer F (2008). Observed sea ice extent in the Russian Arctic, 1933–2006, J. Geophys. Res..

[18] Wyatt MG, Curry JA (2014). Role for Eurasian Arctic shelf sea ice in a secularly varying hemispheric climate signal during the 20th century. Clim Dyn.

[19] Hopsch S, Cohen J, Dethloff K (2012). Analysis of a link between fall Arctic sea ice concentration and atmospheric patterns in the following winter. Tellus A.

[20] Comiso JC, Cavalieri D, Parkinson C, Gloersen P (1997). Passive Microwave Algorithms for Sea Ice Concentrations: A Comparison of Two Techniques. Rem. Sens. of the Environ..

[21] Cavalieri, D. J., Parkinson, C. L., Gloersen, P. & Zwally, H. J. *Sea Ice Concentrations from Nimbus-7 SMMR and DMSP SSM/I-SSMIS Passive Microwave Data*, Version 1.1, last updated 31 Dec 2017. Boulder, Colorado USA NASA National Snow and Ice Data Center Distributed Active Archive Center. updated yearly. Accessed August 2018, 10.5067/8GQ8LZQVL0VL (1996).

[22] Meier WN (2005). Comparison of passive microwave ice concentration algorithm retrievals with AVHRR imagery, in Arctic peripheral seas. IEEE Transactions on Geoscience and Remote Sensing.

[23] Comiso, J. *Bootstrap Sea Ice Concentrations from Nimbus-7 SMMR and DMSP SSM/I-SSMIS*. Version 3.0, last updated 31 December 2017. Boulder, Colorado USA: NASA National Snow and Ice Data Center Distributed Active Archive Center, updated 2017. Accessed August 2018, 10.5067/J6JQLS9EJ5HU (2000).

[24] Titchner Holly A., Rayner Nick A. (2014). The Met Office Hadley Centre sea ice and sea surface temperature data set, version 2: 1. Sea ice concentrations. Journal of Geophysical Research: Atmospheres.

[25] Hwang BJ, Barber DG (2006). Pixel-scale evaluation of SSM/I sea-ice algorithms in the marginal ice zone during early fall freeze-up. Hydrological Processes.

[26] Meier, W. *et al*. NOAA/NSIDC Climate Data Record of Passive Microwave Sea Ice Concentration, Version 3. Boulder, *Colorado USA NSIDC: National Snow and Ice Data Center*. Accessed August 2018, 10.7265/N59P2ZTG (2017).

[27] Chapman, W. L. & Walsh, J. E. Arctic and Southern Ocean Sea Ice Concentrations. Boulder, *Colorado USA NSIDC: National Snow and Ice Data Center*, updated 1996, 10.7265/N5057CVT (1991).

[28] Danish Meteorological Institute (DMI) and NSIDC. Arctic Sea Ice Charts from the Danish Meteorological Institute, 1893–1956. Compiled by V. Underhill and F. Fetterer. Boulder, Colorado USA: National Snow and Ice Data Center. 10.7265/N56D5QXC (2012).

[29] Walsh JE, Chapman WL (2001). Twentieth-century sea ice variations from observational data. Annals of Glaciology.

[30] Kelly, P. M. An Arctic Sea Ice Data Set: 1901–1956. Glaciological Data Report: Workshop on Snow Cover and Sea Ice Data. GD-5 p 101–106. World Data Center A for Glaciology (Snow and Ice). Boulder, CO (1979).

[31] Dedrick KR, Partington K, Van Woert M, Bertoia CA, Benner DUS (2001). National/Naval Ice Center Digital Sea Ice Data and Climatology. Can. J. Remote Sens..

[32] National Ice Center. National Ice Center Arctic sea ice charts and climatologies in gridded format (ed. and compiled by Fetterer, F. & Fowler, C.) Boulder, Colorado USA: National Snow and Ice Data Center, updated 2009, 10.7265/N5X34VDB (2006).

[33] Eisenman I, Meier WN, Norris JR (2014). A spurious jump in the satellite record: has Antarctic sea ice expansion been overestimated?. The Cryosphere.

[34] Ivanova N, Johannessen OM, Pedersen LT, Tonboe RT (2014). Retrieval of Arctic sea ice parameters by satellite passive microwave sensors: a comparison of eleven sea ice concentration algorithms, *IEEE Trans*. Geosci. Remote Sensing.

[35] Bunzel F, Notz D, Baehr J, Muller WA, Frolich K (2016). Seasonal climate forecasts significantly affected by observational uncertainty of Arctic sea ice concentration. Geophysical Research Letters.

[36] Danielson S, Curchitser E, Hedstrom K, Weingartner T, Stabeno P (2011). On ocean and sea ice modes of variability in the Bering Sea. J. Geophys. Res. Oceans.

[37] Bjorgo E, Johannessen OM, Miles MW (1997). Analysis of merged SMMR-SSMI time series of Arctic and Antarctic sea ice parameters 1978–1995. Geophysical Research Letters.

[38] Cavalieri DJ, Parkinson CL, DiGirolamo N, Ivanoff A (2012). Intersensor Calibration Between F13 SSMI and F17 SSMIS for Global Sea Ice Data Records. IEEE Geosci. Remote Sens. Lett..

[39] Comiso, J. C. & Nishio, F. Trends in the sea ice cover using enhanced and compatible AMSR-E, SSM/I, and SMMR data, *Journal of Geophysical Research: Oceans*, **113**(C2), 10.1029/2007JC004257 (2008).

[40] National Snow and Ice Data Center.: http://nsidc.org/the-drift/data-update/noaansidc-releases-sea-ice-index-version-2/available from July 6, 2016.

[41] Niebauer HJ (1998). Variability in Bering Sea ice cover as affected by a regime shift in the North Pacific in the period 1947–1996. Journal of Geophysical Research-Oceans.

[42] Walsh JE, Johnson CM (1979). An analysis of Arctic sea ice fluctuations, 1953–77. J. Phys. Oceanogr..

[43] Close, S., Houssais, M.-N., & Herbaut, C. Regional dependence in the timing of onset of rapid decline in Arctic sea ice concentration, *J*. *Geophys*. *Res*. *Oceans*, **120**, 8077–8098, 10.1002/2015/JC011187.

[44] Holland MM, Bitz CM, Tremblay B (2006). Future abrupt reductions in the summer Arctic sea ice. Geophysical Research Letters.

[45] Tschudi, M., Fowler, C., Maslanik, J., Stewart, J. S. & Meier, W. EASE-Grid Sea Ice Age Boulder, Colorado USA: NASA National Snow and Ice Data Center Distributed Active Archive Center, Version 3, last updated April 8, 2016 and accessed April 12, 2016, 10.5067/PFSVFZA9Y85G (2016).

[46] Lynch AH, Serreze MC, Cassano EN, Crawford AD, Stroeve J (2016). Linkages between Arctic summer circulation regimes and regional sea ice anomalies. J. Geophys. Res..

[47] Rodionov SN (2004). A sequential algorithm for testing climate regime shifts. Geophysical Research Letters.

