# Supplementary material for: The step-like evolution of Arctic open water
Source: Sci Rep. 2018 Nov 15;8:16902. doi: 10.1038/s41598-018-35064-5 (PMC6237816; doi:10.1038/s41598-018-35064-5)
Supplement: Supplementary file 1 — Supplementary Information [file 41598_2018_35064_MOESM1_ESM.doc]

**Supplement for:**

**The step-like evolution of Arctic open water.**

Michael A. Goldstein*^1,2^, Amanda H. Lynch^3,4^, Andras Zsom^5^, Todd Arbetter^3^, Andres Chang^3^, and Florence Fetterer^6^

^1^Climate Change Research Center, University of New South Wales, Sydney NSW 2052 Australia.

^2^Finance Division, Babson College, Babson Park, MA 02457 USA.

^3^Institute at Brown for Environment and Society, Brown University, Providence, RI 02912 USA.

^4^Department of Earth, Environmental and Planetary Sciences, Brown University, Providence, RI 02912 USA.

^5^Data Science Practice, Computing & Information Services, Brown University, Providence, RI 02912 USA.

^6^National Snow and Ice Data Center, Cooperative Institute for Research in the Environmental Sciences, University of Colorado, Boulder, CO 80309 USA.

** Correspondence to*: Michael A. Goldstein (goldstein@babson.edu)

**Panel A: NSIDC-NASA Team**

| **Pacific** |  |  |  |  |  |  |  |  |  |  |  |
| --- | --- | --- | --- | --- | --- | --- | --- | --- | --- | --- | --- |
| p | L [years] | regimes | MSE [km^2] | adj R^2 | BIC | Breakpoint Years | | |  |  |  |
| Linear |  |  | 1.43827E+11 | 0.72788 | 1006.140 |  |  |  |  |  |  |
| 0.05 | 5 | 1 | 1.907E+11 | 0.63919 | 1017.140 |  |  |  |  | 2007 |  |
| 0.05 | 6 | 3 | 1.00561E+11 | 0.80974 | 994.700 | 1989 |  | 2002 |  | 2007 |  |
| 0.05 | 7 | 2 | 1.32761E+11 | 0.74881 | 1004.230 |  |  | 2002 |  | 2007 |  |
| 0.05 | 8 | 1 | 1.71235E+11 | 0.67602 | 1012.940 |  |  | 2002 |  |  |  |
| 0.05 | 9 | 1 | 1.71235E+11 | 0.67602 | 1012.940 |  |  | 2002 |  |  |  |
| 0.05 | 10 | 2 | 1.40999E+11 | 0.73323 | 1006.570 | 1989 |  | 2002 |  |  |  |
|  |  |  |  |  |  |  |  |  |  |  |  |
| **Atlantic** |  |  |  |  |  |  |  |  |  |  |  |
| p | L [years] | regimes | MSE [km^2] | adj R^2 | BIC | Breakpoint Years | | |  |  |  |
| Linear |  |  | 26414277832 | 0.45841 | 940.047 |  |  |  |  |  |  |
| 0.05 | 5 | 0 | 48771276329 | n.m. | 962.844 |  |  |  |  |  |  |
| 0.05 | 6 | 0 | 48771276329 | n.m. | 962.844 |  |  |  |  |  |  |
| 0.05 | 7 | 0 | 48771276329 | n.m. | 962.844 |  |  |  |  |  |  |
| 0.05 | 8 | 1 | 33198676905 | 0.3193 | 948.963 |  |  |  |  |  | 2011 |
| 0.05 | 9 | 1 | 33198676905 | 0.3193 | 948.963 |  |  |  |  |  | 2011 |
| 0.05 | 10 | 1 | 28757744406 | 0.41035 | 943.362 |  | 2008 |  |  |  |  |

| **Canadian** | |  |  |  |  |  |  |  |  |  |
| --- | --- | --- | --- | --- | --- | --- | --- | --- | --- | --- |
| p | L [years] | regimes | MSE [km^2] | adj R^2 | BIC | Breakpoint Years | | |  |  |
| Linear |  |  | 52916225631 | 0.62499 | 967.144 |  |  |  |  |  |
| 0.05 | 5 | 2 | 37194129498 | 0.73641 | 954.602 |  | 1998 |  |  | 2008 |
| 0.05 | 6 | 2 | 37194129498 | 0.73641 | 954.602 |  | 1998 |  |  | 2008 |
| 0.05 | 7 | 2 | 36584652267 | 0.74073 | 953.957 |  | 1998 |  |  | 2007 |
| 0.05 | 8 | 2 | 36584652267 | 0.74073 | 953.957 |  | 1998 |  |  | 2007 |
| 0.05 | 9 | 2 | 37194129498 | 0.73641 | 954.602 |  | 1998 |  |  | 2008 |
| 0.05 | 10 | 2 | 37194129498 | 0.73641 | 954.602 |  | 1998 |  |  | 2008 |
|  |  |  |  |  |  |  |  |  |  |  |
| **Russian** |  |  |  |  |  |  |  |  |  |  |
| p | L [years] | regimes | MSE [km^2] | adj R^2 | BIC | Breakpoint Years | | |  |  |
| Linear |  |  | 84637669571 | 0.72593 | 985.461 |  |  |  |  |  |
| 0.05 | 5 | 2 | 57048637149 | 0.81527 | 971.284 | 1990 |  |  | 2005 |  |
| 0.05 | 6 | 2 | 57048637149 | 0.81527 | 971.284 | 1990 |  |  | 2005 |  |
| 0.05 | 7 | 2 | 57048637149 | 0.81527 | 971.284 | 1990 |  |  | 2005 |  |
| 0.05 | 8 | 2 | 57048637149 | 0.81527 | 971.284 | 1990 |  |  | 2005 |  |
| 0.05 | 9 | 2 | 57048637149 | 0.81527 | 971.284 | 1990 |  |  | 2005 |  |
| 0.05 | 10 | 2 | 57048637149 | 0.81527 | 971.284 | 1990 |  |  | 2005 |  |
|  |  |  |  |  |  |  |  |  |  |  |
| **All Arctic** | |  |  |  |  |  |  |  |  |  |
| p | L [years] | regimes | MSE [km^2] | adj R^2 | BIC | Breakpoint Years | | |  |  |
| Linear |  |  | 1.64598E+11 | 0.81572 | 1011.400 |  |  |  |  |  |
| 0.05 | 5 | 2 | 1.30379E+11 | 0.85404 | 1003.520 |  | 1998 |  |  | 2007 |
| 0.05 | 6 | 3 | 98273159178 | 0.88998 | 993.800 | 1989 | 1998 |  |  | 2007 |
| 0.05 | 7 | 3 | 98273159178 | 0.88998 | 993.800 | 1989 | 1998 |  |  | 2007 |
| 0.05 | 8 | 3 | 98273159178 | 0.88998 | 993.800 | 1989 | 1998 |  |  | 2007 |
| 0.05 | 9 | 3 | 98273159178 | 0.88998 | 993.800 | 1989 | 1998 |  |  | 2007 |
| 0.05 | 10 | 3 | 98273159178 | 0.88998 | 993.800 | 1989 | 1998 |  |  | 2007 |

**Panel B: HAD-Truncated**

| **Pacific** |  |  |  |  |  |  |  |  |  |  |
| --- | --- | --- | --- | --- | --- | --- | --- | --- | --- | --- |
| p | L [years] | regimes | MSE [km^2] | adj R^2 | BIC | Breakpoint Years | | |  |  |
| Linear |  |  | 2.4705E+11 | 0.71719 | 1027.24 |  |  |  |  |  |
| 0.05 | 5 | 1 | 3.0407E+11 | 0.65191 | 1035.34 |  |  |  | 2007 |  |
| 0.05 | 6 | 3 | 1.6333E+11 | 0.81302 | 1013.61 | 1989 | 2002 |  | 2007 |  |
| 0.05 | 7 | 3 | 1.6333E+11 | 0.81302 | 1013.61 | 1989 | 2002 |  | 2007 |  |
| 0.05 | 8 | 1 | 2.1203E+11 | 0.75727 | 1022.49 |  | 2002 |  | 2007 |  |
| 0.05 | 9 | 1 | 2.8089E+11 | 0.67844 | 1032.25 |  | 2002 |  |  |  |
| 0.05 | 10 | 2 | 2.3546E+11 | 0.73046 | 1026.57 | 1989 | 2002 |  |  |  |
|  |  |  |  |  |  |  |  |  |  |  |
| **Atlantic** |  |  |  |  |  |  |  |  |  |  |
| p | L [years] | regimes | MSE [km^2] | adj R^2 | BIC | Breakpoint Years | | |  |  |
| Linear |  |  | 5.2651E+10 | 0.60139 | 966.95 |  |  |  |  |  |
| 0.05 | 5 | 0 | 1.3208E+11 | n.m. | 1001.7 |  |  |  |  |  |
| 0.05 | 6 | 0 | 1.3208E+11 | n.m. | 1001.7 |  |  |  |  |  |
| 0.05 | 7 | 0 | 1.3208E+11 | n.m. | 1001.7 |  |  |  |  |  |
| 0.05 | 8 | 2 | 4.8059E+10 | 0.63615 | 964.596 |  | 2000 |  |  | 2011 |
| 0.05 | 9 | 2 | 4.8059E+10 | 0.63615 | 964.596 |  | 2000 |  |  | 2011 |
| 0.05 | 10 | 2 | 4.8059E+10 | 0.63615 | 964.596 |  | 2000 |  |  | 2011 |

| **Canadian** |  |  |  |  |  |  |  |  |  |  |
| --- | --- | --- | --- | --- | --- | --- | --- | --- | --- | --- |
| p | L [years] | regimes | MSE [km^2] | adj R^2 | BIC | Breakpoint Years | | |  |  |
| Linear |  |  | 7.9738E+10 | 0.70710 | 983.136 |  |  |  |  |  |
| 0.05 | 5 | 2 | 5.1248E+10 | 0.81175 | 967.102 |  | 1998 |  | 2007 |  |
| 0.05 | 6 | 2 | 5.1248E+10 | 0.81175 | 967.102 |  | 1998 |  | 2007 |  |
| 0.05 | 7 | 2 | 5.1248E+10 | 0.81175 | 967.102 |  | 1998 |  | 2007 |  |
| 0.05 | 8 | 2 | 5.1248E+10 | 0.81175 | 967.102 |  | 1998 |  | 2007 |  |
| 0.05 | 9 | 2 | 5.1248E+10 | 0.81175 | 967.102 |  | 1998 |  | 2007 |  |
| 0.05 | 10 | 2 | 5.1248E+10 | 0.81175 | 967.102 |  | 1998 |  | 2007 |  |
|  |  |  |  |  |  |  |  |  |  |  |
| **Russian** |  |  |  |  |  |  |  |  |  |  |
| p | L [years] | regimes | MSE [km^2] | adj R^2 | BIC | Breakpoint Years | | |  |  |
| Linear |  |  | 1.7306E+11 | 0.69983 | 1013.36 |  |  |  |  |  |
| 0.05 | 5 | 1 | 1.5734E+11 | 0.72708 | 1009.64 |  |  | 2005 |  |  |
| 0.05 | 6 | 1 | 1.5734E+11 | 0.72708 | 1009.64 |  |  | 2005 |  |  |
| 0.05 | 7 | 1 | 1.5734E+11 | 0.72708 | 1009.64 |  |  | 2005 |  |  |
| 0.05 | 8 | 1 | 1.5734E+11 | 0.72708 | 1009.64 |  |  | 2005 |  |  |
| 0.05 | 9 | 1 | 1.5734E+11 | 0.72708 | 1009.64 |  |  | 2005 |  |  |
| 0.05 | 10 | 1 | 1.5734E+11 | 0.72708 | 1009.64 |  |  | 2005 |  |  |
|  |  |  |  |  |  |  |  |  |  |  |
| **All Arctic** |  |  |  |  |  |  |  |  |  |  |
| p | L [years] | regimes | MSE [km^2] | adj R^2 | BIC | Breakpoint Years | | |  |  |
| Linear |  |  | 3.3987E+11 | 0.77830 | 1039.68 |  |  |  |  |  |
| 0.05 | 5 | 1 | 4.6384E+11 | 0.69743 | 1051.81 |  |  |  | 2007 |  |
| 0.05 | 6 | 2 | 2.9971E+11 | 0.80449 | 1035.98 |  | 2002 |  | 2007 |  |
| 0.05 | 7 | 2 | 2.9971E+11 | 0.80449 | 1035.98 |  | 2002 |  | 2007 |  |
| 0.05 | 8 | 2 | 2.9971E+11 | 0.80449 | 1035.98 |  | 2002 |  | 2007 |  |
| 0.05 | 9 | 2 | 2.9971E+11 | 0.80449 | 1035.98 |  | 2002 |  | 2007 |  |
| 0.05 | 10 | 2 | 3.7006E+11 | 0.75860 | 1044.21 |  | 2002 |  |  | 2010 |

**Panel C: HAD-Full**

| **Pacific** |  |  |  |  |  |  |  |  |  |  |  |
| --- | --- | --- | --- | --- | --- | --- | --- | --- | --- | --- | --- |
| p | L [years] | regimes | MSE [km^2] | adj R^2 | BIC | Breakpoint Years | | |  |  |  |
| Linear |  |  | 3.549E+11 | 0.56497 | 1546.62 |  |  |  |  |  |  |
| 0.05 | 5 | 1 | 3.072E+11 | 0.62345 | 1538.25 |  |  |  |  | 2007 |  |
| 0.05 | 6 | 3 | 1.91E+11 | 0.76594 | 1513.00 |  | 1989 | 2002 |  | 2007 |  |
| 0.05 | 7 | 3 | 1.91E+11 | 0.76594 | 1513.00 |  | 1989 | 2002 |  | 2007 |  |
| 0.05 | 8 | 2 | 2.272E+11 | 0.72145 | 1521.90 |  |  | 2002 |  | 2007 |  |
| 0.05 | 9 | 2 | 2.272E+11 | 0.72145 | 1521.90 |  |  | 2002 |  | 2007 |  |
| 0.05 | 10 | 2 | 2.377E+11 | 0.70869 | 1524.50 |  | 1989 | 2002 |  |  |  |
|  |  |  |  |  |  |  |  |  |  |  |  |
| **Atlantic** |  |  |  |  |  |  |  |  |  |  |  |
| p | L [years] | regimes | MSE [km^2] | adj R^2 | BIC | Breakpoint Years | | |  |  |  |
| Linear |  |  | 6.017E+10 | 0.60529 | 1443.69 |  |  |  |  |  |  |
| 0.05 | 5 | 1 | 1.235E+11 | 0.18999 | 1485.39 | 1971 |  |  |  |  |  |
| 0.05 | 6 | 0 | 1.524E+11 | n.m. | 1496.53 |  |  |  |  |  |  |
| 0.05 | 7 | 1 | 1.235E+11 | 0.18999 | 1485.39 | 1971 |  |  |  |  |  |
| 0.05 | 8 | 3 | 4.967E+10 | 0.67418 | 1434.90 | 1971 |  | 2000 |  |  | 2011 |
| 0.05 | 9 | 2 | 5.58E+10 | 0.63396 | 1440.45 |  |  | 2000 |  |  | 2011 |
| 0.05 | 10 | 2 | 5.58E+10 | 0.63396 | 1440.45 |  |  | 2000 |  |  | 2011 |

| **Canadian** |  |  |  |  |  |  |  |  |  |  |
| --- | --- | --- | --- | --- | --- | --- | --- | --- | --- | --- |
| p | L [years] | regimes | MSE [km^2] | adj R^2 | BIC | Breakpoint Years | | |  |  |
| Linear |  |  | 1.342E+11 | 0.45921 | 1490.21 |  |  |  |  |  |
| 0.05 | 5 | 2 | 6.488E+10 | 0.73853 | 1449.20 |  |  | 1998 |  | 2007 |
| 0.05 | 6 | 2 | 6.488E+10 | 0.73853 | 1449.20 |  |  | 1998 |  | 2007 |
| 0.05 | 7 | 2 | 6.488E+10 | 0.73853 | 1449.20 |  |  | 1998 |  | 2007 |
| 0.05 | 8 | 2 | 6.488E+10 | 0.73853 | 1449.20 |  |  | 1998 |  | 2007 |
| 0.05 | 9 | 2 | 6.488E+10 | 0.73853 | 1449.20 |  |  | 1998 |  | 2007 |
| 0.05 | 10 | 2 | 6.488E+10 | 0.73853 | 1449.20 |  |  | 1998 |  | 2007 |
|  |  |  |  |  |  |  |  |  |  |  |
| **Russian** |  |  |  |  |  |  |  |  |  |  |
| p | L [years] | regimes | MSE [km^2] | adj R^2 | BIC | Breakpoint Years | | |  |  |
| Linear |  |  | 1.973E+11 | 0.67846 | 1512.58 |  |  |  |  |  |
| 0.05 | 5 | 2 | 1.319E+11 | 0.78515 | 1490.33 |  | 1990 |  | 2005 |  |
| 0.05 | 6 | 2 | 1.425E+11 | 0.76784 | 1494.82 | 1971 |  |  | 2005 |  |
| 0.05 | 7 | 2 | 1.425E+11 | 0.76784 | 1494.82 | 1971 |  |  | 2005 |  |
| 0.05 | 8 | 3 | 1.137E+11 | 0.81472 | 1482.94 | 1971 | 1990 |  | 2005 |  |
| 0.05 | 9 | 2 | 1.425E+11 | 0.76784 | 1494.82 | 1971 |  |  | 2005 |  |
| 0.05 | 10 | 3 | 1.137E+11 | 0.81472 | 1482.94 | 1971 | 1990 |  | 2005 |  |
|  |  |  |  |  |  |  |  |  |  |  |
| **All Arctic** |  |  |  |  |  |  |  |  |  |  |
| p | L [years] |  | MSE [km^2] | adj R^2 | BIC | Breakpoint Years | | |  |  |
| Linear |  |  | 5.214E+11 | 0.65665 | 1568.93 |  |  |  |  |  |
| 0.05 | 5 | 1 | 5.072E+11 | 0.66594 | 1567.34 |  |  |  |  | 2007 |
| 0.05 | 6 | 2 | 3.456E+11 | 0.77243 | 1546.21 |  |  |  | 2002 | 2007 |
| 0.05 | 7 | 2 | 3.082E+11 | 0.79702 | 1539.58 |  |  | 1999 |  | 2007 |
| 0.05 | 8 | 2 | 3.456E+11 | 0.77243 | 1546.21 |  |  |  | 2002 | 2007 |
| 0.05 | 9 | 2 | 3.456E+11 | 0.77243 | 1546.21 |  |  |  | 2002 | 2007 |
| 0.05 | 10 | 2 | 2.965E+11 | 0.80476 | 1537.32 |  |  | 1998 |  | 2007 |

**Supplement Table 1. Mean Square Error (MSE), Adjusted R^2^ (Adj R^2) and Bayesian Information Criterion (BIC) for linear and breakpoint models, along with the breakpoint years for the Atlantic, Pacific, Canadian, and Russian sectors, and for the whole Arctic, for different year lengths in the sequential algorithm with p=0.05. The data for Panel A is from the NSIDC-NASA Team open water record for 1979-2017; Panel B, the HadISST record for 1979-2017; and Panel C, the HadISST record for 1953-2017.**
